# From oppression to opportunity: a pilot study of an intervention program for vulnerable first generation college students

**DOI:** 10.3389/fpsyg.2023.1149746

**Published:** 2023-06-20

**Authors:** Ma. Teresa Tuason, Lynne Carroll, Marina Schutz, Sara Buchanan

**Affiliations:** ^1^Department of Public Health, University of North Florida, Jacksonville, FL, United States; ^2^Department of Psychology, University of North Florida, Jacksonville, FL, United States; ^3^Department of Psychology, Otto-Friedrich-Universität Bamberg, Bamberg, Bavaria, Germany

**Keywords:** socio-demographic vulnerabilities, intervention program, first-generation college students, financial struggles, medical-mental illness, self-efficacy, college adaptation

## Abstract

The purpose of this pilot study was to examine the impact of a 10-week psycho-educational group intervention entitled “Oppression to Opportunity Program” (OOP), which was designed to enhance the academic adjustment of vulnerable, first-generation college students. Participants in the pilot group also experienced multiplicative vulnerabilities as result of the intersecting identities of race, ethnicity, income, religious affiliation, disabilities, sexual orientation, and gender identity. The OOP intervention consisted of eight modules, plus an orientation and a closing session, designed to lessen key barriers (e.g., lack of knowledge of resources, lack of access to high quality mentorships, feelings of isolation) to academic success. The modules incorporated written worksheets and experiential exercises to foster group discussion, participant self-reflection, and community belongingness. Each group met once weekly for 1 h each session over 10 weeks, and was facilitated by an advanced graduate student in counseling. Participants completed the College Self-Efficacy Inventory and Student Adaptation to College Questionnaire as a pretest and post-test, and qualitative after session questionnaires. MANOVA results did not demonstrate a significant difference between OOP (*n* = 30) and comparison group (*n* = 33) undergraduates on efficacy and student adaptation. However, ANCOVA results show the impact of group (OOP vs. comparison) on post-tests of self-efficacy and adaptation, while controlling for pre-tests as covariates. Male participants rated the module pertaining to goal setting and establishing role models as most favorable, while the emotional management module was most preferred by female participants. African American participants rated the module pertaining to identity affirmation as most beneficial and the emotional management module was most preferable for Hispanic Americans. Lastly, Caucasian Americans rated the module on finding and sustaining supportive relationships as most favorable. Preliminary results were promising, however, the OOP program needs to be replicated in larger samples. Recommendations were offered including lessons learned regarding challenges associated with the implementation of a pre-post non-equivalent group design. Lastly, the significance of being flexible while building a sense of community, and the importance of providing food, supportive counseling, and peer mentoring were emphasized.

## Introduction

Postsecondary education is associated with considerable benefits. However, not every college student has an equal chance to successfully attend and graduate from college (Ishitani, 2006). Prior research demonstrates that particular groups of students possess a higher risk of dropping out of college (e.g., Billson and Terry, 1982; Martinez et al., 2009). The purpose of this pilot study was to examine the impact of a psychoeducational group intervention entitled Oppression to Opportunity Program (OOP). OOP was designed to promote vulnerable first-generation students’ academic self-efficacy and their adaptation to academic life. Vulnerability is defined as “an identifiable increased likelihood of incurring additional or greater wrong” (Hurst, 2008, p. 195). Intersecting factors of race, ethnicity, income, religious affiliation, psychiatric and physical disabilities, sexual orientation and gender identity expose first-generation undergraduates to multiplicative vulnerabilities in terms of academic self-efficacy and academic performance. The current study includes participants who experience these vulnerabilities in various ways as they attempt to adjust to the new demands of being in college. The primary purpose of the Oppression to Opportunity Program (OOP) was to aid first-generation college students in their transition to college life. First generation students, defined as students whose parents have not completed an undergraduate degree (Hurst, 2008), are a population proven to be at risk of dropping out of college (e.g., Billson and Terry, 1982; Martinez et al., 2009). The personal identity characteristics (i.e., physical disability, ethnicity, sexual orientation, etc.,) contribute to students’ difficulties in reaching their academic goals. Participants of the program were “doubly disadvantaged;” a population of students who are low-income minority students and who graduated from “under-resourced” public high schools and who know little about the implicit norms and aspects of college life (Jack, 2019). This paper details the design and implementation of the OOP and the results of a pilot study which was undertaken with the first cohort of program participants. The pilot study operationalized academic adjustment as a combination of academic self-efficacy and academic adaptation including course success and social success. Academic self-efficacy is defined as the belief in one’s ability to successfully attain one’s academic goals. A high self-efficacy is significantly correlated with academic success (Solberg et al., 1993).

### First-generation college students, vulnerable students and their barriers

Researchers (e.g., Billson and Terry, 1982) investigated the barriers students face, specifically the phenomenon of attrition, of why certain students persist in college and others drop out of college prior to graduation. Additionally, Skinner and Richardson (1988) reported two dimensions, preparation for college and perceived relationship between education and opportunity, such that students may be more likely to succeed if they are well prepared and perceive their education as a useful tool to boost their vocational development. Piorkowski (1983) considered another barrier to flourishing in college that some students who come from low-income families and live in urban areas were facing, the so-called “survivor guilt” (p. 620). He used this term to describe emotional and psychosocial problems students face as they encounter criticism from family or as they question themselves given that they have the opportunity to attend college. In their cultural mismatch theory, Stephens et al. (2012) called attention to another possible explanation for the educational disadvantage of first-generation college students and the resulting social class achievement gap. This implied that first-generation students are exposed to a cultural mismatch, as they are used to identifying themselves as being part of, and depending on a community (interdependence), whereas in college, they encounter a learning and performance environment which requires them to primarily rely on themselves (independence).

As a result of all these, researchers (e.g., Garcia, 2010; Stephens et al., 2012) stressed the need for interventions addressing cultural obstacles that lead to the achievement gap. Additionally, once vulnerable students are enrolled in college, it is also important to understand how to motivate them intrinsically and extrinsically in these intervention programs, since motivation is crucial for academic success and college completion (Petty, 2014). Factors that are thought to be associated with attrition are the motivation to obtain a college degree, substance use, perceived challenges in college, and psychological distress (Martinez et al., 2009). However, these factors were not found to mediate the relation between low parental education and attrition but do predict attrition (Martinez et al., 2009). This indicates it is still worth having a closer look at how parents influence their children’s academic career (Gibbons and Woodside, 2014).

### Proposed innovation for vulnerable college students

This 10-week intervention consisted of eight modules, plus an orientation and closing session. These modules were based on previous research findings (e.g., Bryan and Simmons, 2009) which identified key barriers to academic success experienced by ethnic and racial minority students (i.e., lack of knowledge of resources and access to high quality mentorships, feelings of isolation, etc.). The modules were designed to incorporate important information on each topic that they discuss their experiences in a safe space with each other. Worksheets and experiential exercises were designed by the first author to foster self-reflection and build community and belongingness with each other. Advanced graduate students in counseling served as facilitators. Each group met once weekly for 1 h each session (at the same time in the afternoon, on the same day of the week) over 10 weeks. The facilitators introduced the selected topic, administered worksheets and facilitated the small group discussions that took place.

#### Establishing identity

Cross (1971) pointed out the need for the educational system to adjust in order to allow first-generation college students, who are overrepresented by Black Americans, Mexican Americans, and Native Americans, to realize their full potential. As we identified these vulnerabilities, we spent time in this module identifying students’ self-expectations and who they wanted to become. With identities characterized by lacking a sense of communal identity (Orbe, 2004) and perceiving less support from their families (York-Anderson and Bowman, 1991), we facilitated the discussion of their process of navigating through college and figuring out elements to their identity, especially preserving their identification with college, an element in the Futures Project intervention by Saunders and Serna (2004) with first-generation Hispanic students.

#### Keeping supportive relationships

Vulnerable, first-time college students face hardships in their social adjustment to college (Salzer, 2012). The small group community format utilized in the OPP afforded students with opportunities to voice their many concerns about college, including financial problems and academic struggles, and receive advise and support from the facilitators and other students. In addition, participants were encouraged to nurture those relationships throughout several weeks of the term. The community structure of the OPP intervention is similar in this way to the Freshmen Empowerment program (FEP; Folger et al., 2004).

#### Taking care of my academics

Because different degrees of vulnerabilities pose academic difficulties, students may become less involved in campus life and consequently may have lower graduation rates (Salzer, 2012). They often encounter complexities that are due to poor quality of prior education (Nora and Rendon, 1996). Especially for first generation college students, their parents cannot share knowledge about college preparation so an unfortunate consequence is difficulty with college retention (Martinez et al., 2009). Programs with disadvantaged students (e.g., Ronald E. McNair Postbaccalaureate Achievement Program) have found that providing access to research experiences, academic counseling, mentoring, and attending research internships prepared students for graduate school and provided students with increased motivation to strive for advanced degrees and make a career in higher education (U.S. Department of Education, 2007; Gittens, 2014). In this intervention, we discussed time management, in terms of devoting enough time to study, while working and keeping a healthy support system. During this session, facilitators shared detailed information about several campus resources including academic advising services, the writing and study skill center, career and mental health counseling centers, the child-care center, and the computer lab. Lastly, facilitators shared their own experiences engaging with these resources, seeking learning opportunities such as internships and developing mentoring relationships with professors.

#### Taking care of my physical health

Because vulnerabilities included in the study pertained to physical and mental illnesses, we devoted one module on the extent by which students currently nurture their health. We discussed behaviors pertaining to: nutrition and food intake (i.e., the diet they were used to in their families, food that is comforting for them, and food that makes them healthier), health risks in their families (i.e., illnesses, causes of death, and ways of coping with stress), sleep hygiene (i.e., the amount of sleep they get versus what they need), and level of physical activity for pleasure, strength, and stamina. We helped them identify their goals and motivations for overall physical and mental health and referred them to the Counseling and Student Health Centers.

#### Taking care of my money

A module on financial literacy was included in the OOP because vulnerable, first generation college students are often economically disadvantaged. Poverty has a negative impact on colleges success (Horn, 2007). Moreover, reflecting on students’ experiences of their socio-economic status in their family while growing up and their family’s habits of handling money are important considerations. We discussed students’ current financial pressures, their issues about balancing their checkbook, budgeting, financial aid, and transportation, and the importance of living within their means, because it has been found that among students with vulnerabilities, most are financially independent (Engle and Tinto, 2008).

#### Managing emotions

Because regulating emotions are vital to building close relationships (English et al., 2013), we discussed ways by which students deal with their emotions. In this module, we helped students identify their most frequently experienced emotions, emotions that are foreign to them, and those that they avoid. Students conversed about the ways they have been aware of their emotions and how they experience, cope, and express these. We also talked how these emotions are related to their behavior, thoughts, and self-care.

#### Taking care of my mental health and family issues

Because vulnerabilities of students may lead them to encounter stigmatization by faculty and peers due to unawareness or incomprehension (Loewen, 1993), we talked openly about mental health issues. We talked about the diagnoses they or their families have lived with and their understanding of how these affect them in college. Not only students’ parents (Gibbons and Woodside, 2014), but also their extended families and communities were shown to have an impact on college success (Bryan and Simmons, 2009). Thereby, we discussed their familial histories and we guided them into constructing their family genograms (McGoldrick et al., 2008), to be able to identify the generational patterns in their families of origin and be able to take responsibility for their choices, behaviors, personalities, and future.

#### My role models and my dreams

Because previous research (e.g., Nora and Rendon, 1996) reported that vulnerable, first generation students drop out of college, in part, because of a lack sufficient role models and a college-related commitment, we deemed it important to focus on identifying people in students’ lives that they could look up to and serve as an example for them. We also conducted a career visualization exercise, to help students project into the future, and envision the kind of life and career they would like for themselves. An intervention called Young Scholars Program (Newman and Newman, 1999), had the same aim of increasing minority students’ persistence. Resilience research has shown that those who have dreams set for themselves and who have had expectations of them, become more hardy vis-à-vis difficult life circumstances (Masten, 2001).

### Impact on self-efficacy and college success

Self-efficacy was defined by Bandura (1997) as “one’s confidence in their ability to control their emotions, behaviors, and actions in order to actualize desired objectives” (as cited in Wood et al., 2015, p. 3). In social cognitive theory, Bandura stressed the impact of self-efficacy on all aspects of goal formation and achievement. In this intervention, college self-efficacy was assessed as an outcome variable because of established relationships between self-efficacy beliefs with student adjustment (Chemers et al., 2001), with academic success and degree persistence (Robbins et al., 2004), and with academic success of minority students (Fuertes and Sedlacek, 1995; Majer, 2009). Moreover, the malleability of self-efficacy was shown in several studies (e.g., Mathisen and Bronnick, 2009), and was increased through mentoring (Santos and Reigadas, 2002). Thus, the OOP was developed as a tool to foster college students’ self-efficacy because it involved mentoring and support from a community of peers.

### Impact on adjustment to college

Meta-analytic evidence suggests that adjustment to college is a multidimensional construct which predicts college grades and college retention substantially (Credé and Niehorster, 2012). In light of high rates of college attrition (Engle and Theokas, 2010), understanding the relationship between adjustment to college and retention is crucial (Credé and Niehorster, 2012). The OOP aims at increasing students’ adaptation to college by providing mentoring and skills training, information about available resources, offering support in small group communities.

Thus, the goal of this study was to assess the effectiveness of the OOP that is designed to promote a sense of community among vulnerable first-generation students, and examine the impact on their academic self-efficacy and adaptation to college. We compared members of the intervention group compared to an equivalent group, and compared the intervention group with themselves before and after the program, as used in previous studies (e.g., Vuong et al., 2010).

## Context in which the innovation occurs

### Setting and participants

Participants of the intervention group were recruited from university offices and referred by staff personnel who provided services to students with vulnerabilities that increase their risk of dropping out of college. Participants were also recruited from first year classes through announcements in person and using campus media outlets. Although 30 students attended the initial informational session, only 16 remained and were each assigned to smaller intervention communities. Moreover, although we had recruited and advertised for freshmen and sophomores only, juniors and seniors were interested in the intervention and volunteered to participate in the study. The second cohort of 14 students received the same intervention in the following semester. These two cohorts will be referred to as one intervention group, n = 30. Further, in a next semester, another group comprising of 33 college students in two classes were assessed as a comparison. Students were initially asked to participate in the intervention program, but because none of them were interested in participating, they were asked to volunteer as the comparison group.

Vulnerable students were self-identified, and when asked to rate their domain(s) of vulnerability on a scale from 0 (*not having much impact*) to 10 (*having much impact*), participants reported that the vulnerabilities that had the most impact on them (reported 7 and above on the Likert scale from 1–10), in descending order, were: being first-generation college students (43.6%), having mental illness in their families (43.6%), having medical illness in their families (38.8%), being affected by poverty (34%), being an ethnic minority (31.2%), one’s own mental illness (30.7%), their age (27.4%), belonging to a sexual minority (19.7%), one’s own medical illness (16.1%), and being a religious minority (14.8%).

The participants in the intervention program have an age range of 17–61 years old, with a mean of 24.33 and *SD* = 9.81. Females comprise 76.7, and 65.5% identify as poor. They are mostly heterosexuals, 83.3%, and U.S.-born, 82.1%. They are comprised of 40% African Americans, 30% Caucasians, 23.3% Hispanic Americans, and 3.3% Asian Americans. On the other hand, the participants that did not participate in the intervention program have an age range of 19–26 years old, with a mean of 22.76 and *SD* = 3.47. Females comprise 72.7%, and only 25% identify as poor. They are mostly heterosexuals, 78.8%, and U.S.-born, 87.9%. They are comprised of 78% Caucasians, 9.4% Hispanic Americans, 9.4% Asian Americans, and 3.1% African Americans.

## Detail to understand key programmatic elements

A community case study of comparing 2 groups was used to measure the effectiveness of the 10-week intervention which covered the following topics: Establishing identity, Keeping supportive relationships, Taking care of my academics, Taking care of my physical health, Taking care of my money, Managing emotions, Taking care of my mental health and family issues, and My role models and my dreams. The weekly, one-hour sessions took place in 5 small groups, consisting of the same 3 to 6 students across 10-weeks, and led by the same group facilitor. These small groups served as the community of peers whose vulnerabilities were similar to theirs, and who they met regularly exploring parts of their selves together. Refreshments were provided at each weekly session. Participants reported that this was helpful for those on a strict budget and whose schedules often mandated these missed meals. Five graduate students enrolled in the university’s Master’s program in Mental Health Counseling served as group facilitators. Facilitators participated in an OPP facilitator training session before the commencement of the program and in weekly one-hour supervision sessions.

Pre-and post intervention program data was collected, and in the following year, students who had received the intervention were offered the opportunity to participate in a bi-monthly support follow-up sessions in their groups. These sessions allowed them to reunite with their community of peers, consult on their needs and concerns that arise, and gave them continued support even after the project.

### Measures

A consent form was given along with a demographic questionnaire that asked participants about their gender, sexual orientation, age, country of birth, ethnicity, religion, vulnerabilities, first language, emigration, family position, children in their family, primary caretaker, marital status, own children, current income level and their high school GPA. Approval was obtained from the University of North Florida Institutional Review Board with approval #680592–1.

#### College self-efficacy inventory

The College Self-Efficacy Inventory (Solberg et al., 1993) is comprised of 19 items referring to three domains of college life concerning courses, roommates, and social situations. Each subscale score is the mean of the responses on a nine-point Likert-type scale ranging from 0 (*totally unconfident*) to 8 (*totally confident*). Overall CSEI scores are the sum of the responses on all items, where higher scores depict higher college self-efficacy. Internal consistency reliability estimates were α = 0.93 for the overall CSEI and α = 0.88 for each of the three subscales, indicating strong internal consistency reliability (Solberg et al., 1993). Moreover, the CSEI was found to be robust to differences in acculturation, gender, or class level with good convergent and discriminant validity. In the current study, Cronbach’s alpha for the overall CSEI scale was α = 0.83 before the intervention program and α = 0.92 after the intervention program. The internal consistency reliability estimates for course, roommate, and social efficacy were α = 0.83, 0.73, and 0.83 before the intervention, and α = 0.82, 0.62, and 0.87 after the intervention. An additional reliability index for internal consistency, the Composite Reliability (CR) for the course, roommate, and social efficacy were 0.63, 0.73, and 0.73 before the intervention, and 0.55, 0.31, and 0.67 for after the intervention. Furthermore, the Average Variance Extracted (AVE), a measure establishing convergent validity for the course, roommate, and social were 0.26, 0.44, and 0.30 before the intervention, and 0.25, 0.27, and 0.25 for after the intervention---all showing inadequate convergent validity.

#### Student adaptation to college questionnaire

The 67-item Student Adaptation to College Questionnaire (SACQ; Baker and Siryk, 1984, 1987) was used to assess college adjustment. It measures four facets of adjustment: academic, social, personal-emotional, and attachment. The overall SACQ score is the mean of students’ responses based on all 67 items. Each subscale score is the mean of the responses on a nine-point Likert-type scale ranging from 1 (*does not apply to me at all*) to 9 (*applies very closely to me*). Higher scores were indicative of higher self-perceived adaptation to college. Internal consistency reliability coefficients in a study with first-generation college students were obtained by Baker and Siryk (1987): α = 0.83 to 0.89 for the academic subscale, α = 0.83 to 0.91 for the social subscale, α =0.77 to 0.85 for the personal-emotional subscale, and α = 0.85 to 0.91 for the attachment subscale. In the current study, α = 0.94 for the overall SACQ before the intervention and α = 0.98 after the intervention. For the four subscales, α = 0.83 (academic), 0.87 (social), 0.84 (personal-emotional), and 0.73 (attachment) before the intervention program and α = 0.90 (academic), 0.96 (social), 0.76 (personal-emotional) and 0.83 (social) after the intervention. An additional reliability index for internal consistency, the Composite Reliability (CR) for the academic, social, personal-emotional and attachment adjustment were 0.96, 0.97, 0.89, and 0.93 after the intervention. Furthermore, the Average Variance Extracted (AVE), a measure establishing convergent validity for the academic, social, personal-emotional and attachment adjustment were 0.64, 0.66, 0.58 and 0.60 after the intervention---all showing adequate convergent validity.

#### Weekly after session questionnaire

Participants completed a written post-session evaluation at the end of each weekly session. This measure was modified from prior research (Neumeister and Rinker, 2006). Participants rated on a 5-point Likert scale (0 = least to 5 most) their level of interest in, and usefulness of, the module topic for that session and the degree to which the session supported them to “believe in their dreams.”

### Data analysis

Multivariate Analysis of Variance (MANOVA) was conducted to assess the differences between the intervention and the comparison group on college self-efficacy and adaptation. Analysis of Covariance (ANCOVA) was conducted to assess the impact on post-test college self-efficacy and adaptation of group (intervention vs. comparison) while controlling for the pre-tests as covariates. After session questionnaire data were analyzed in terms of comparing means, and frequencies thru Chi Square analyses.

## Results

### Preliminary analyses

Means, standard deviations, and bivariate correlations between variables (CSEI and SACQ; demographics) are reported for the intervention group (see Table 1) and for the comparison group (see Table 2). For the intervention group, correlation analyses show significant negative associations between age and course efficacy (i.e., older participants have lower course self-efficacy) and significant positive association between income level and overall SACQ (i.e., students with higher income were more adapted to college). Further, income level correlate positively with overall CSEI, for both intervention and control groups (i.e., students with higher income have higher levels of college self-efficacy).

**Table 1:** Means, standard deviations, and correlations between study variables –intervention group.

Measure	*M*	*SD*	1	2	3	4	5	6	7	8	9	10	11	12	13	14	15	16	17	18	19	20
1. Age	24.33	9.81	—	−0.36	−0.32	−0.35	−0.46^*^	−0.13	−0.10	−0.02	−0.08	−0.49	−0.10	−0.18	−0.10	−0.19	−0.12	−0.19	−0.03	−0.28	−0.02	0.12
2. Income level^d^	3.17	1.79		—	0.45^*^	0.15	0.19	−0.25	0.26	0.43	0.44^*^	0.28	0.54^**^	0.33	0.21	0.20	0.61^**^	0.39	0.50^**^	0.14	0.43^*^	0.07
3. CSEI	5.51	0.96			—	0.67^*^	0.70^**^	0.31	0.48^**^	0.36	0.77^**^	0.73^**^	0.73^**^	0.43	0.53^**^	0.18	0.64^**^	0.58^*^	0.55^**^	0.45	0.56^**^	0.32
4. CSEI P	6.62	0.67				—	0.80^**^	0.80^**^	−0.11	0.36	0.52	0.87^**^	0.44	0.50	0.48	0.48	0.44	0.49^*^	−0.04	0.11	0.54	0.50
5. C-SE	5.47	1.31					—	0.74^**^	0.12	0.09	0.24	0.68^*^	0.52^**^	0.57^*^	0.67^**^	0.52	0.20	0.53	0.34	0.48	0.36	0.40
6. C-SE P	6.44	0.94						—	−0.35	−0.01	0.02	0.48	0.50	0.31	0.71^**^	0.43	0.20	0.19	0.02	0.12	0.56^*^	0.29
7. R-SE	5.73	1.65							—	0.35	0.09	−0.03	0.28	0.08	−0.05	−0.24	0.28	0.11	0.46^*^	0.62^*^	0.06	−0.09
8. R-SE P	6.69	0.80								—	0.38	0.20	−0.33	0.18	−0.21	0.06	−0.00	0.31	−0.34	0.16	−0.31	0.08
9. S-SE	5.42	1.40									—	0.72^**^	0.59^**^	0.16	0.34	−0.07	0.71^**^	0.49	0.33	−0.06	0.58^**^	0.26
10. S-SE P	6.74	0.90										—	0.46	0.51	0.28	0.41	0.59^*^	0.71^**^	0.06	0.01	0.58^*^	0.58^*^
11. SACQ	5.79	0.95											—	0.26	0.73^**^	0.34	0.79^**^	0.25	0.83^**^	0.10	0.81^**^	0.23
12. SACQ P	6.41	0.96												—	0.31	0.92^**^	0.14	0.88^**^	0.07	0.59^**^	0.39	0.82^**^
13. AA SACQ	5.97	1.01													—	0.54	0.33	0.22	0.44^*^	0.04	0.49^**^	0.20
14. AA SACQ P	6.71	1.05														—	0.05	0.74^**^	−0.10	0.41	0.37	0.70^**^
15. SA SACQ	5.68	1.40															—	.0.38	0.57^**^	−0.18	0.76^**^	0.24
16. SA SACQ P	6.54	1.13																—	−0.06	0.32	0.41	0.87^**^
17. PE SACQ	4.74	1.59																	—	0.34	0.56^**^	−0.02
18. PE SACQ P	5.15	1.39																		—	−0.03	0.17
19. A SACQ	6.74	1.08																			—	0.51
20. A SACQ P	6.83	1.42																				—

CSEI, College Self-Efficacy Inventory; CSEI P, College Self-Efficacy Inventory Post; C-SE, Course Self-Efficacy; C-SE P, Course Self-Efficacy Post; R-SE, Roommate Self-Efficacy; R-SE P, Roommate Self-Efficacy Post; S-SE, Social Self-Efficacy; S-SE P, Social Self-Efficacy Post; SACQ, Student Adaptation to College Questionnaire; SACQ P, Student Adaptation to College Questionnaire Post; AA SACQ, Academic Adjustment SACQ; AA SACQ P, Academic Adjustment SACQ Post; SA SACQ, Social Adjustment SACQ; SA SACQ P, Social Adjustment SACQ Post; PE SACQ P, Personal-Emotional SACQ Post; A SACQ, Attachment SACQ; A SACQ P, Attachment SACQ.

Income level was rated on a 10-point Likert scale ranging from 1 (*poor*) to 10 (*rich*).

^*^p < 0.05. ^**^p < 0.01.

**Table 2:** Means, standard deviations, and correlations between study variables –comparison group.

Measure	*M*	*SD*	1	2	3	4	5	6	7	8	9	10	11
1. Age	22.76	3.47	—	0.24	−0.05	−0.20	−0.10	0.08	−0.0	−0.07	−0.03	0.06	0.01
2. Income level	4.72	2.05		—	0.46^**^	0.37^*^	0.38^*^	0.47^**^	0.31	0.38^*^	0.14	0.16	0.26
3. CSEI	5.58	1.27			—	0.90^**^	0.87^**^	0.96^**^	0.37^*^	0.42^*^	0.22	0.13	0.33
4. Course SE	5.69	1.23				—	0.72^**^	0.75^**^	0.34	0.50^**^	0.07	0.15	0.21
5. Roommate SE	6.04	1.17					—	0.79^**^	0.24	0.24	16	0.08	0.27
6. Social SE	5.25	1.62						—	0.38^*^	0.37^*^	0.30	0.12	0.38^*^
7. SACQ	5.86	1.33							—	0.86^**^	0.73^**^	0.69^**^	0.77^**^
8. AA SACQ	5.90	1.33								—	0.43^*^	0.50^**^	0.58^**^
9. SA SACQ	6.06	1.31									—	0.33	0.56^**^
10. PE SACQ	5.06	1.71										—	0.17
11. A SACQ	6.47	1.49											—

CSEI, College Self-Efficacy Inventory; Course SE, Course Self-Efficacy; Roommate SE, Roommate Self-Efficacy; Social SE, Social Self-Efficacy; SACQ, Student Adaptation to College Questionnaire; AA SACQ, Academic Adjustment SACQ; SA SACQ, Social Adjustment SACQ; PE SACQ, Personal-Emotional SACQ; A SACQ, Attachment SACQ.

Income level was rated on a 10-point Likert scale ranging from 1 (*poor*) to 10 (*rich*).

^*^p < 0.05. ^**^p < 0.01.

Focusing on correlations between the scales among all participants, a significant correlation was found between CSEI and SACQ scales, and the social efficacy subscale had the highest correlation with overall CSEI. The personal-emotional subscale had the highest correlation with overall SACQ for the intervention group, and the academic adjustment subscale had the highest correlation with overall SACQ for the control group.

### Comparisons between intervention and comparison groups

Because of limitations due to logistics and time, there was a lack of a true comparison group with real before and after intervention data. The data was collected at only one time with the comparison group and as such it is unknown whether the comparison group is truly equivalent to the intervention group. It is important to note this limitation in data collection, in the following comparisons reported.

With MANOVA analyses, the assumption of equality of covariance matrices has been met using the Box’s test of Equality of Covariance, *p* = 0.44; the assumption of sphericity has been met and is useful with repeated-measures design, *p* < 0.001; the Levene’s test of equality are all non significant pointing to a robust analysis. Pillai’s trace is used as accurate because group sizes are unequal, covariance matrices seem homogeneous, and assuming multivariate normality is reasonable (Field, 2013). MANOVA results show (not including CSEI and SACQ total scores), there was no significant effect of being in the intervention and control groups on CSEI and SACQ subscales, Pillai’s trace *V* = 0.27, *F*(7,37) = 1.94, *p* = 0.09. However, separate univariate ANOVAs on the outcome variables revealed significant treatment effects on Post Social Efficacy scores, *F*(1, 43) = 9.69, *p* = 0.003.

### Comparisons between before and after intervention

**Figure 1:**
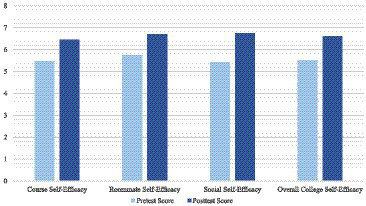
A comparison of college self-efficacy mean scores measured before the intervention and after the intervention.

**Figure 2:**
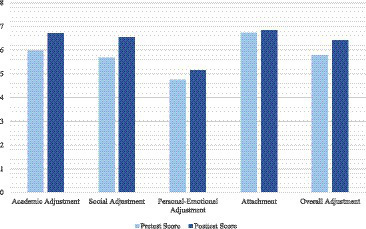
A comparison of adaptation to college mean scores measured before the intervention and after the intervention.

Focusing on the intervention group alone, means differ between pre and post intervention scores for the total college self-efficacy score and course, roommate, and social self efficacy as seen in Figure 1. Means between pre and post intervention scores also differ in the overall adaptation to college score and academic, social, personal-emotional, and attachment as seen in Figure 2. With ANCOVA analyses, Levene’s test is significant, *p*’s < 0.001, violating the homogeneity of variance. When doing the ANCOVA tests, with pre-tests as covariates and post-tests as dependent variables, the covariate, Pre Academic Adjustment SACQ, was significantly related to Post Academic Adjustment SACQ, *F*(1, 35) = 13,592, *p* < 0.001, *r^2^* = 0.87; Pre Social Adjustment SACQ, was significantly related to Post Social Adjustment SACQ, *F*(1, 35) = 17,925, *p* < 0.001, *r^2^* = 0.78; Pre Personal Emotional Adjustment SACQ, was significantly related to Post Personal Emotional Adjustment SACQ, *F*(1, 35) = 9,824, *p* < 0.001, *r^2^* = 0.82; Pre Attachment Adjustment SACQ, was significantly related to Post Attachment Adjustment SACQ, *F*(1, 35) = 26,230, *p* < 0.001, *r^2^* = 0.82.

Additionally, there was also a significant effect on group (intervention vs. control) on post-tests controlling for the effect of pre-tests (covariates). These scores are in Table 3.

**Table 3:** Effects of group (Intervention vs. Comparison) on the post-tests after controlling for the effects of pre-tests as covariates with bootstrap analyses.

DV	Covariate	*F* (1, 45,035)	*r^2^*
Post Total CSEI	Pre total CSEI	84020^***^	0.95
Post Course SE	Pre course SE	42183^***^	0.89
Post Roommate SE	Pre roommate SE	8361^***^	0.77
Post Social SE	Pre social SE	86117^***^	0.96
Post Total SACQ	Pre total SACQ	14196^***^	0.80
Post Academic SACQ	Pre AA SACQ	20641^***^	0.87
Post Social SACQ	Pre social SACQ	10118^***^	0.78
Post Personal-Emotional SACQ	Post personal-emotional SACQ	18350^***^	0.82

CSEI, College Self-Efficacy Inventory; Course SE, Course Self-Efficacy; Roommate SE, Roommate Self-Efficacy; Social SE, Social Self-Efficacy; SACQ, Student Adaptation to College Questionnaire; Academic SACQ, Academic Adjustment SACQ; Social SACQ, Social Adjustment SACQ; Per-Emo SACQ, Personal-Emotional SACQ; A SACQ, Attachment SACQ.

*** *p* < 0.001.

In summary, when pre-test scores are controlled for, there is significant impact of group (whether intervention or control) on the post-tests.

### Session summaries

Post-intervention questionnaire responses (ranging from 1 *low* to 5 *high*) were analyzed by comparing means. Out of the 10-week intervention sessions, participants found the session on Role Models and Dreams to be the most helpful (*M* = 4.69; *SD* = 0.48) and the most interesting (*M* = 4.77*; SD* = 0.44). Managing Emotions helped them feel group support (*M* = 4.83 *SD* = 0.41) and belongingness (*M* = 4.83 *SD* = 0.41). To participants, Taking care of Academics was the least helpful (*M* = 3.50 *SD* = 1.2), the least interesting (*M* = 3.71 *SD* = 1.3), and felt least belongingness to the group (*M* = 4.00 *SD* = 1.1). The session on taking care of my Mental Health and Family Issues was the least related to feeling group support (*M* = 4.33 *SD* = 0.87).

In terms of favorability (helpfulness, interest, college help, group support, group belongingness, belief in one’s dreams), males found the session on role models and pursuing one’s dreams to be the most favorable (χ = 4.60) and the session on taking care of academics to be the least favorable (χ = 3.62); while females found the session on managing emotions to be the most favorable (χ = 4.58), and the session on taking care of mental health and family issues to be the least favorable (χ = 3.57).

In terms of favorability (helpfulness, interest, college help, group support, group belongingness, belief in one’s dreams), African Americans found the session on establishing one’s identity to be the most favorable (χ = 4.58) and the session on taking care of one’s mental health and family issues to be the least favorable (χ = 3.28). Hispanic Americans found the session on managing emotions to be the most favorable (χ = 4.89) and the session on keeping supportive relationships to be the least favorable (χ = 3.87). Caucasian Americans found the session on keeping supportive relationships to be the most favorable (χ = 4.57) and the session on taking care of academics to be the least favorable (χ = 3.40). Asian Americans were not included in the analyses because there was only one Asian American participant in the intervention group.

An examination of students’ evaluations of the entire intervention (session summaries) revealed that participants gave their highest ratings on the statements about feeling supported by the group, feeling that they belonged to the group and that the sessions were interesting to them. This indicates that a strong group cohesion, a sense of community with peers who were also vulnerable like they are, was established through the weekly group meetings and that the chosen topics were of relevance to the vulnerable students.

## Discussion

This pilot study sought to explore participants’ responses to a 10-week psychoeducational intervention which was designed to increase vulnerable, first-generation college students academic self-efficacy and adaptation to academic life, thru enhancing a sense of community. We found promising preliminary support for the use of the OOP intervention. A larger, more representative sample is needed to provide further confirmation of the effectiveness of the OOP intervention. Correlation analyses showed that older students reported lower levels of college self-efficacy, and students with higher income reported higher levels of college self-efficacy and perceived themselves as more adapted to college. These findings are in line with the assumption that poverty is a risk factor for college students (e.g., Bryan and Simmons, 2009).

MANOVA analyses did not show significant effects on the overall differences between the intervention and comparison groups. However, ANCOVA analyses showed that while controlling for pre test scores as covariates, being on the intervention and comparison groups had significant impact on both self-efficacy and student adaptation to college. These significant differences were seen in before and after scores in all three dimensions of college self-efficacy: course, roommate, and social efficacy and most dimensions of college adjustment as well: academic, social, and personal-emotional adjustment with moderately large to large effect sizes. In sum, the intervention successfully fostered self-efficacy and student adaptation, which provides further support for the malleability of these factors relevant to college functioning (e.g., Mathisen and Bronnick, 2009).

The session theme on identifying role models and dreams is deemed most helpful and interesting---a finding that supports previous research that mentoring increases college self-efficacy and positively influences adjustment to college, perceived mentor supportiveness, and program satisfaction (Santos and Reigadas, 2002). Managing Emotions is important to feeling belongingness and support by the group, both being integral mechanisms for college adjustment (Dennis et al., 2005), as well as a positive influence on mental health outcomes (Stephens et al., 2014). Males liked discussing role models and pursuing one’s dreams, while females liked managing emotions. African Americans liked discussing establishing one’s identity; Hispanic Americans liked discussing managing emotions; Caucasian Americans liked talking about keeping supportive relationships.

### Practical implications for higher education

Lessons learned from this intervention may serve to better guide practitioners’ efforts to provide vulnerable college students with peer support that is vital to psychosocial development (Credé and Niehorster, 2012), with psychoeducation that fosters well-being and mental health among college students (Higginbotham, 2013), and with counseling that is significantly related to college retention (Lee et al., 2009). First, time commitment must be required and expected in interventions that equip vulnerable individuals with the psychosocial resources to flourish at college. It is quite beneficial to have these interventions required (course credit), more focused, containing the necessary themes, but provided in 2–4 weeks, of 1 h session weekly, as opposed to 8–10 weeks. Being in a small group of 4 people like the one conducted in the study, will provide enough time for processing and an adequate number of group members to experience groupness, universality, and belongingness. This structure may serve to motivate vulnerable students, particularly for academic success and college completion (Petty, 2014).

For future intervention research measuring college self-efficacy and adaptation to college, it may be valuable not to exclusively rely on self-report data through including objective measures of college functioning. Moreover, it may be more practical to obtain follow-up data using technology, such as with a survey link online. Also, a comparison group, that contains positive elements such as journal-keeping or a brief supportive psychotherapy group may be more appropriate to ascertain outcome for intervention research in this field.

### Acknowledgment of methodological constraints

The study was carried out, with limitations and challenges that inform future community studies on intervention feasibility. There was great difficulty in getting consistent voluntary participation for 10 weeks, due to students’ hectic schedules (i.e., work, family obligations, transportation issues), making the groups really small. Such attrition is not uncommon for such a purposive sample of first-generation college students with self-identified vulnerabilities (Martinez et al., 2009), but the time commitment for a 10-week intervention exacerbated this attrition rate. The group attendance fluctuated between the sessions over the course of the study.

Moreover, an investigator-driven efficacy study was planned with true wait list control group, but it was difficult to carry out logistically, due to the expressed immediate need for support by the members of the initial control group. With this initial wait-list control group dissolved, we resorted to having a group just for comparison, but with the limitation that students’ college self-efficacy and adaptation to college could only be measured once. It would have increased interpretability and rigor if a real control group was measured before and after an interval of time, even when no intervention was received.

Our observations also showed that building a sense of community in their small groups was a powerful incentive for increasing attendance. We shifted gears and instead of focusing on the rigor of the intervention, we highlighted the camaraderie, the community of same-ness, and universality of the members of the groups.

Because students with vulnerabilities have had to start from disadvantaged positions, counseling interventions do not only need to be more persevering, they also need to be effective, so as to give vulnerable students the chance to believe in themselves, flourish to their potential, and aspire for a promising future. More importantly, they needed to be given in small groups, so as to give vulnerable students a sense of community and to powerfully believe that they are not alone.

## Data availability statement

The raw data supporting the conclusions of this article will be made available by the authors, without undue reservation.

## Ethics statement

The studies involving human participants were reviewed and approved by University of North Florida IRB, approval # 680592–1. The patients/participants provided their written informed consent to participate in this study.

## Author contributions

MT: conceptualization of the work, research design, data collection, analysis, and revising the written work. LC: data collection, data analysis and interpretation, drafting the work, and critical revision. MS: data analysis, data interpretation, and drafting the written work. SB: drafting the work and critical revision. All authors contributed to the article and approved the submitted version.

## Conflict of interest

The authors declare that the research was conducted in the absence of any commercial or financial relationships that could be construed as a potential conflict of interest.

## Publisher’s note

All claims expressed in this article are solely those of the authors and do not necessarily represent those of their affiliated organizations, or those of the publisher, the editors and the reviewers. Any product that may be evaluated in this article, or claim that may be made by its manufacturer, is not guaranteed or endorsed by the publisher.
